# Pathogenesis and therapeutic opportunities of gut microbiome dysbiosis in critical illness

**DOI:** 10.1080/19490976.2024.2351478

**Published:** 2024-05-23

**Authors:** Nicole A Cho, Kathryn Strayer, Breenna Dobson, Braedon McDonald

**Affiliations:** a Department of Critical Care Medicine, Cumming School of Medicine, University of Calgary, Calgary, AB, Canada; b Snyder Institute for Chronic Diseases, Cumming School of Medicine, University of Calgary, Calgary, AB, Canada

**Keywords:** Intensive care, critical care, critical illness, microbiome, host-microbe interaction, hospital-acquired infections, nosocomial infections, ventilator-associated pneumonia

## Abstract

For many years, it has been hypothesized that pathological changes to the gut microbiome in critical illness is a driver of infections, organ dysfunction, and other adverse outcomes in the intensive care unit (ICU). The advent of contemporary microbiome methodologies and multi-omics tools have allowed researchers to test this hypothesis by dissecting host–microbe interactions in the gut to better define its contribution to critical illness pathogenesis. Observational studies of patients in ICUs have revealed that gut microbial communities are profoundly altered in critical illness, characterized by markedly reduced alpha diversity, loss of commensal taxa, and expansion of potential pathogens. These key features of ICU gut dysbiosis have been associated with adverse outcomes including life-threatening hospital-acquired (nosocomial) infections. Current research strives to define cellular and molecular mechanisms connecting gut dysbiosis with infections and other outcomes, and to identify opportunities for therapeutic modulation of host–microbe interactions. This review synthesizes evidence from studies of critically ill patients that have informed our understanding of intestinal dysbiosis in the ICU, mechanisms linking dysbiosis to infections and other adverse outcomes, as well as clinical trials of microbiota-modifying therapies. Additionally, we discuss novel avenues for precision microbial therapeutics to combat nosocomial infections and other life-threatening complications of critical illness.

## 1. Introduction

Critically ill patients suffer from life-threatening conditions, including sepsis, trauma, respiratory failure, and others that require life-support interventions in intensive care units (ICUs). Patients with critical illness often suffer from multiple-organ failure, extreme physiologic stress, and a high susceptibility to hospital-acquired (nosocomial) infections, which all culminate in a high risk of mortality,^1–3^ as well as long-term physical, cognitive, and psychiatric sequelae in survivors.^4^ Among the organ systems affected in critical illness, emerging evidence has revealed that the microbial communities of the intestinal tract experiences profound ecological and functional disturbances.^5^ Importantly, like injury to other organ systems (cardiovascular, pulmonary, renal, etc.), dysfunction of the gut microbiota in critical illness is associated with major adverse clinical outcomes including death, prolonged life support, and life-threatening nosocomial infections.^5–8^ This association between intestinal dysbiosis and adverse outcomes has led to the hypothesis that therapeutic correction of intestinal microbial communities may represent a new avenue for life-support therapy in the ICU.^9^ A crucial next step in the development of microbiome-targeted interventions for critical illness is to build upon these observational associations, toward a causal and mechanistic understanding of how gut microbes contribute to pathogenesis, to identify rationalized and effective therapeutic targets.

Critically ill patients demonstrate severe microbiota dysbiosis across multiple body sites, with the gut being the most extensively studied to date. A recent systematic review and meta-analysis highlighted core features of early gut dysbiosis in critical illness that is characterized by depletion of anaerobes that are prominent commensals in the healthy gut including *Bifidobacterium, Blautia*, and *Faecalibacterium*, coupled with expansion and overabundance of putative pathogens including *Enterococcaceae* and *Enterobacteriaceae*.^6^ This review will summarize the prominent ecological and functional changes of the gut microbiota in critical illness, and explore recent advances in our understanding of mechanisms linking dysbiosis to adverse clinical outcomes in the ICU including nosocomial infections, and how these mechanistic insights may be targeted therapeutically.

## 2. Gut microbiome dysbiosis in critical illness

**Figure 1.**
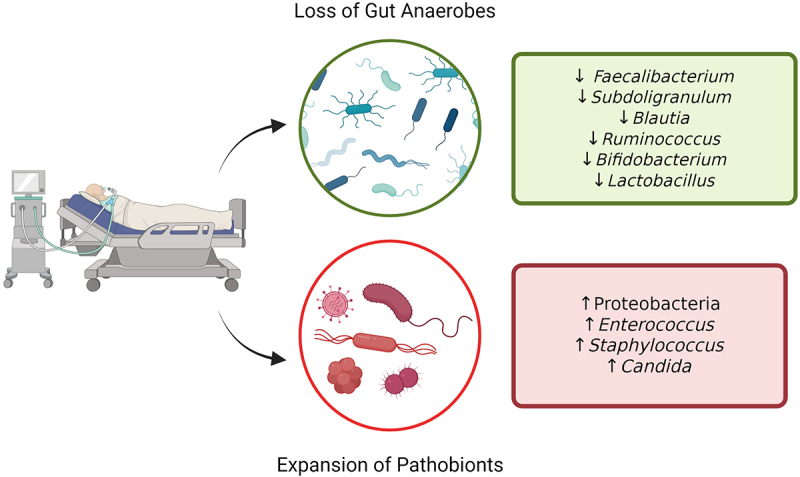
Gut microbiota dysbiosis in critical illness. Hallmark features of ICU gut dysbiosis include reduction of community alpha diversity, associated with the loss of commensal anaerobes, and expansion of pathobionts. Created in BioRender.com.

The gut microbiome of ICU patients undergoes extreme compositional and functional changes compared to healthy individuals (Figure 1). Hallmarks of this ICU dysbiosis include reduced alpha diversity, reduction of commensal taxa that are abundant in the healthy gut, and overgrowth of organisms with pathogenic potential (pathobionts).^6^ For the purpose of this review, the term “dysbiosis” will broadly encompass altered microbial composition associated with disease state, which may include loss of colonization resistance, pathobiont expansion, or both, as well as other changes to the composition and function of the microbiota that contribute to disease pathogenesis. Taxa such as *Enterococcaceae, Staphylococcus*, and *Enterobacteriaceae* are found in the healthy gut at very low levels and display little to no pathogenicity under homeostasis.^10,11^ In contrast, sequencing analysis of fecal samples from ICU patients often demonstrates that 50% or more of sequence reads are assigned to individual bacterial or fungal species including *Enterococcus faecium*, *Escherichia coli*, *Candida albicans*, or other pathobiont taxa.^12^ These pathobionts are increased in both relative abundance and total biomass, indicating that the gut microenvironment in critical illness favors their growth.^12,13^ Importantly, this prominent expansion of pathobionts in the gut of critically ill patients has been linked, both directly and indirectly, with adverse outcomes including infections and increased mortality, as discussed in more detail below.

In addition to the expansion of potentially pathogenic organisms, another hallmark of ICU dysbiosis is the corresponding loss of commensal members of a healthy gut community. Studies of critically ill patients have consistently found reductions in the relative abundance of key obligate anaerobes, notably the Firmicutes and Bacteroidetes phyla.^5,9,14^ The loss of anaerobic fermenters leads to a reduction in beneficial microbial metabolites such as short-chain fatty acids (SCFAs) like butyrate, which is known to maintain epithelial barrier integrity and contribute to immune homeostasis in the gut.^15^ In critical illness, there is a notable loss of key butyrate-producing genera *Faecalibacterium* and *Subdoligranulum*.^5,16^ In addition, multiple studies have reported reduced *Bifidobacterium* abundance, a taxa that plays an important role in maintaining immune regulation and nutrient absorption, and when analyzed in combination with severity of illness the loss of this genera was associated with increased risk of death in a medical ICU.^7^

Given the prominent changes in gut ecology that appear to characterize ICU dysbiosis and its associations with adverse outcomes, studies have begun to investigate the factors that drive these multi-taxa changes in the gut when people become critically ill. Most notably, alterations in gut microbial communities are attributed to the widespread use of broad-spectrum antibiotics in the ICU. Antibiotic administration in the ICU is commonplace, as demonstrated by the most recent iteration of a worldwide point-prevalence study of infections and antibiotic administration in 15,165 ICU patients, which found 79% of patients received antibiotics (either prophylactically or therapeutically).^1^ Indeed, studies of healthy subjects have confirmed that systemic administration of broad-spectrum antibiotics drives marked changes in microbial ecology in the intestine, including depletion of commensal anaerobes akin to what is observed in ICU patients.^17^ The use of multi-variable analyses in cohort studies of ICU patients has suggested that systemic antimicrobial exposure is an important variable driving microbiota dysbiosis.^18–21^ In addition, comparisons between ICU patients who received antibiotics versus those who did not have also demonstrated an impact on microbiome composition. For example, a prospective observational study of septic and non-septic ICU patients found that antibiotic treatment (in septic patients) was associated with variation in microbiome composition, a reduction in SCFAs (purportedly due to the reduction in anaerobic fermenters), and increased fungal burden.^22^ In addition to systemic antibiotic treatment, prophylactic anti-bacterial and anti-fungal administration for the goal of “selective digestive decontamination” (SDD) is also practiced in some jurisdictions, which will be discussed further below. However, it is important to note that gut microbiota dysbiosis is also observed in patients who have not received antimicrobial treatments (such as those admitted with neurological emergencies, trauma, or other noninfectious diagnoses), and that pathological dysbiosis has been reported in many patients at the time of presentation to the ICU (often within only hours of the inciting illness) prior to treatment with antibiotics.^5,9,23–26^ Therefore, although aggressive antimicrobial exposure surely contributes to gut dysbiosis in the ICU, there are clearly additional drivers of the extreme shifts in microbial communities in this patient population.

**Figure 2.**
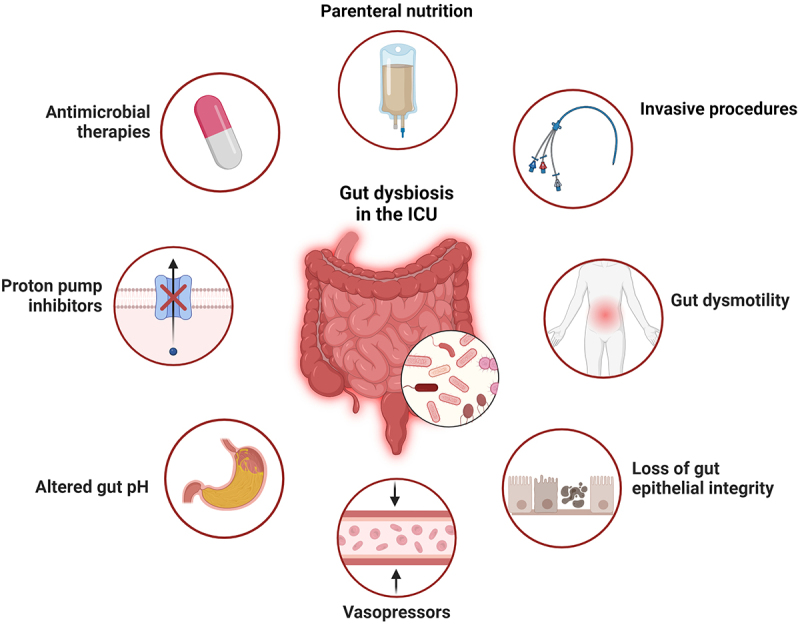
Factors contributing to dysbiosis in critical illness. Multiple factors contribute to the significant shifts observed in microbial ecology of the gut during critical illness, including host intrinsic factors (circulatory shock, systemic acidosis, altered mucosal blood flow, inflammation, and others), as well as host extrinsic influences (antibiotics, nutritional alterations, medications, invasive devices, and others). Created in BioRender.com.

In addition to antibiotics, critically ill patients are exposed to a variety of interventions that may impact gut bacteria and contribute to dysbiosis. For example, gastric acid suppression with medications like proton-pump inhibitors (PPI) are widely used for gastric ulcer prophylaxis in mechanically ventilated patients. Alteration of the upper GI pH with PPI therapy is known to induce microbiota alterations, and use of these medications has been associated with higher rates of dysbiosis-related complications including *C. difficile* infection and ventilator-associated pneumonia (VAP).^27,28^ In addition, profound alterations in nutritional intake that occur during critical illness likely also contribute to alterations in gut microbial ecology. Many patients in the ICU experience periods of time without nutrition, while others receive exclusively intravenous nutrition. Furthermore, enteral nutrition in the ICU is far from normal, as it is administered in the form of partially digested formula via a gastric tube. Coupled with the presence of invasive devices, and the ICU environment from which pathogens (and other microbes) can be acquired, these host-extrinsic factors impart significant selective pressure on microbes of the gut and contribute to ICU dysbiosis (Figure 2).

There are also a variety of host-intrinsic factors during critical illness that contribute to intestinal dysbiosis. Gastrointestinal dysmotility is present in up to 70% of ICU patients, and manifests as a spectrum from impaired motility (impaired gastrointestinal transit (IGT), ileus, acute colonic pseudo-obstruction), to diarrhea. Risk factors for developing GI dysmotility in the ICU include sepsis, mechanical ventilation, and the use of vasopressors, opioids, or anticholinergic medications.^29^ Dysmotility and slowed transit time can result in small intestinal bacterial overgrowth (SIBO) syndrome,^30^ which was found to affect up to 36.5% of patients in a small observational study of critically ill patients.^31^ Overall, gastrointestinal dysmotility presents a challenge in the context of critical illness, as it can worsen dysbiosis, impact nutrient absorption, exacerbates bacterial translocation or overgrowth, and contributes to increased systemic inflammation.^32,33^ Beyond the gut, systemic physiological derangements in critical illness may also exert an important influence on bacterial communities. The presence of circulatory shock, and associated treatment with vasopressors, alters mucosal blood flow and impacts epithelial integrity.^34^ Systemic inflammation associated with critical illness can also impact the gut microenvironment, and gut mucosal inflammation in animal models has been shown to generate a selective advantage for overgrowth of pathobionts such as *Enterobacteriaceae*.^35^

Finally, aside from host-microbe interactions, it is likely that altered microbe–microbe interactions contribute to ICU dysbiosis. In particular, it is hypothesized that breakdown of colonization resistance (CR) mechanisms contributes to pathobiont expansion during critical illness.^36^ Notably, the co-occurrence of anaerobic fermenter depletion with *Enterobacteriaceae* expansion is suggestive of disrupted homeostatic mechanisms of CR. Indeed, abundant preclinical evidence has unraveled important roles for anaerobic fermenters in the suppression of *Enterobacteriaceae* in the gut through production of SCFA, reduction of luminal pH, maintenance of anaerobiosis, and suppression of inflammation which all antagonize growth of *Enterobacteriaceae.*^37–39^ Indeed, a recent study of critically ill patients found that treatment with anti-anaerobe antibiotics was associated with expansion of *Enterobacteriaceae* in gut, possibly due to the disruption of anaerobe-mediated CR mechanisms.^26^ Other common ICU pathobionts such as *Enterococcus* spp. are influenced by disrupted CR during acute illness. For example, in individuals who received hematopoietic stem cell transplant (HSCT) for hematologic malignancies, antibiotic therapy is associated with intestinal overgrowth by vancomycin-resistant *Enterococcus* (VRE), a common cause of nosocomial infections.^40,41^ VRE overgrowth in this patient population was associated with reduced production of a VRE-suppressing antimicrobial peptide (lantibiotic) by the microbiota. Restoring the production of this lantibiotic in the gut of mice using a defined microbial consortium restored colonization resistance against VRE.^41,42^ Further studies are needed to determine the role of specific mechanisms of CR within the ICU microbiome of critically ill patients, but these intriguing observations suggest the possibility that therapeutic strategies aimed at reconstitution of beneficial anaerobes and restoration of colonization resistance may suppress pathobiont overgrowth and reduce adverse outcomes.

## 3. Consequences of gut dysbiosis in critical illness

Gut dysbiosis has been associated with adverse outcomes including increased mortality, duration of life support, duration of hospitalization, and severity of illness.^5,7,8,18^ There have been recent advances in our understanding of mechanisms driving these associations between microbiome dysbiosis and adverse clinical outcomes, including their impact on multi-organ failure, and perhaps most importantly, the role of gut dysbiosis in the development of hospital-acquired (nosocomial) infections. In this section, we will review the literature on intestinal dysbiosis and key adverse outcomes in the ICU including multi-organ dysfunction, and hospital-acquired infections.

### 3.1. Dysbiosis and hospital-acquired (nosocomial) infections in critical illness

**Figure 3.**
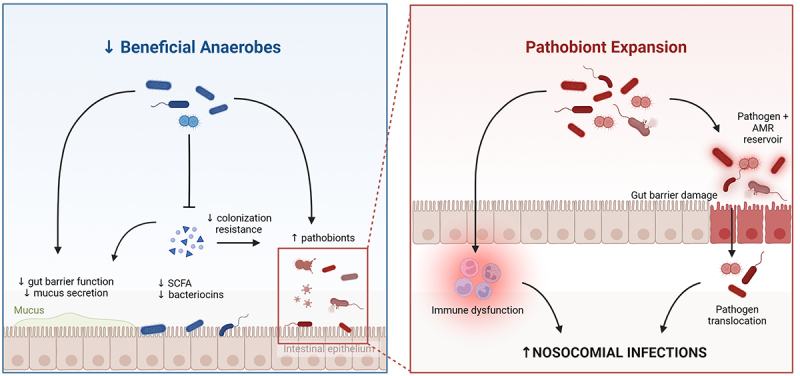
Mechanisms linking gut microbiota dysbiosis with hospital-acquired (nosocomial) infections in critical illness. The loss of commensal anaerobes contributes to breakdown of gut barrier integrity, as well as impaired colonization resistance against pathobiont taxa. Expansion of pathobionts (eg. *Enterobacteriaceae*), coupled with impaired epithelial barrier integrity and systemic immune dysfunction, enables the development of invasive infections by both gut pathobionts (through translocation and dissemination) as well as other pathogens due to impaired systemic immune defense. Created in BioRender.com.

Emerging evidence links gut dysbiosis with the development of deadly hospital-acquired (nosocomial) infections and adverse outcomes in critical illness (Figure 3). Multiple factors contribute to the risk of infections in the ICU, including the breach of physical barriers (intravascular catheters, bladder catheters, endotracheal intubation) and impaired immune defenses, but recent studies have highlighted the gut microbiota as a putative source and driver of nosocomial infections. Observational studies have reported associations between alterations of gut ecology and nosocomial infections, including community metrics such as reduced alpha diversity, as well as specific taxa that are associated with a risk of secondary infections.^43^ The appreciation of microbiota dysbiosis as a contributor to infections in the ICU has prompted researchers to begin translating these correlations into causal linkages, identifying pathogenic mechanisms, and seeking novel treatment targets for rationalized microbiota modification therapies. In this section, we will discuss emerging evidence around the mechanisms linking gut dysbiosis to infection in the ICU.

*The gut as a pathogen reservoir in the ICU –* As described above, a hallmark feature of the ICU gut microbiome is overrepresentation of potentially pathogenic organisms that are usually at low abundance or absent from the healthy gut. For decades, it has been appreciated that there is prominent overlap between the pathobionts that dominate the gut of ICU patients, and the microbiology of pathogens that are cultured from sites of infection – most commonly lung infections in patients on mechanical ventilation (VAP), and/or bloodstream infections.^1,44–46^ The application of culture-independent analysis methods in this patient population has confirmed that among the vast changes in microbial ecology in ICU patients, the presence and/or dominance of certain pathobiont taxa in the gut is associated with higher incidences of extra-intestinal infections by the same taxa, such as *Enterobacteriaceae* and *Enterococcus* spp. that commonly dominate acutely-ill patients.^25,40,47–52^ These findings suggest that the dysbiotic microbiota of critical illness contributes to hospital-acquired infections, in part, through the emergence of a pathogen reservoir in the gut. In response to a breakdown of gut barrier integrity and dysregulation of immune defenses, this reservoir of pathogens may translocate out of the gut and disseminate to other body compartments to cause invasive infections. In addition to translocation and dissemination of whole pathogens, gut dysbiosis and permeability may contribute to systemic organ damage and dysfunction through translocation of microbial products and metabolites that exacerbate systemic inflammation and end-organ injury.

Translocation of pathogens out of the gut lumen and into the blood or lymphatic channels as a route of systemic infection has been well documented in animal models.^53–55^ In humans, establishing gut translocation as the source of a systemic infections (such as bloodstream infections or pneumonia) is much more complicated and painstaking. Early evidence of this was observed in patients with hematologic malignancies undergoing hematopoietic stem cell transplantation (HSCT). Pre-transplant conditioning regimens for allogeneic HSCT often includes radiotherapy, chemotherapy, and antibiotics leading to suppressed host immunity, mucosal barrier injury, and intestinal dysbiosis, culminating in a high risk of bloodstream infections (BSI).^40,41,56^ Analyses of the gut microbiota in combination with BSI pathogens in HSCT patients revealed that intestinal domination by *Enterococcus* (defined in these studies as at least 30% relative abundance) was associated with a 9-fold increased risk of vancomycin-resistant *Enterococcus* bacteremia.^40^ Even stronger associations with BSI have been reported for intestinal domination by Gram-negative pathobionts including *E. coli*, *Klebsiella* spp, *Enterobacter* ssp., *Stenotrophomonas* spp., and *Citrobacter* spp.^49^ These data suggested that BSIin HSCT patients may be preceded by intestinal overgrowth of the same organism, providing indirect evidence for translocation as a causal mechanism linking gut dysbiosis to nosocomial infections.^40^ Similar relationships have been identified between gut colonization by *Candida* spp. and the risk of candidemia in HSCT patients.^57^ In a study of patients with severe COVID-19 (>50% were admitted to ICU), individuals with bloodstream infections had an associated enrichment of the same genera of bacteria in their gut together with depletion of *Faecalibacterium prauznitzii*, suggesting that secondary bacterial bloodstream infections in COVID-19 may also be linked to pathobiont translocation from the gut.^50^ To further define causal relationships between gut dysbiosis and bloodstream infections, multiple studies have conducted painstaking molecular detective work using metagenomic strain-tracking approaches to convincingly demonstrate that BSI pathogens originate from the gut microbiota (as opposed to other potential sources such as the ICU environment). In HSCT recipients with bloodstream infections, Tamburini and colleagues developed a bioinformatic StrainSifter tool to demonstrate that bloodstream infections caused by *E. coli* and *Klebsiella pneumoniae* could be traced back to the patient’s gut microbiota as the likely source.^48^ Genomic analysis revealed that BSI and gut metagenome-assembled genomes (MAGs) of infecting pathogen strains within the same patient were more closely related than strains between different patients, indicating an individual-specific source of infection.^48^ In addition, a recent study of critically ill neonates with bloodstream infections found that more than half patients harbored the identical (or nearly identical) pathogen strain in their gut microbiota prior to infection, with genomic similarity of <20 nucleotide differences.^51^ These rigorous nucleotide-level comparisons of infecting pathogens and gut-derived MAGs provided the best evidence that some bloodstream infections in acutely ill and critically ill patients originate from translocation of pathobionts from the gut microbiota into the bloodstream.

Despite the methodological challenges of source tracking pathogens in the blood, this is more easily performed for bloodstream infections that tend to be monomicrobial and have a logical and direct anatomical route for translocation. In contrast, the more complex microbiology of other nosocomial infections, such as pneumonia in the lung, are more difficult to directly link to the gut as a source of pathogens. Indeed, the clinical microbiology of hospital- and ventilator-associated pneumonia reveals an important role for Gram-negative pathogens that are classically gut-associated.^1^ However, the lung harbors its own bona fide microbiome which itself demonstrates severe dysbiosis during critical illness.^58^ How the dysbiosis in the lung relates to dysbiosis of the gut is incompletely understood. Multiple studies have reported the accumulation of putatively gut-derived microbes in the lung microbiota in patients who develop nosocomial pneumonia,^58–60^ as well as patients with acute respiratory distress syndrome (ARDS).^58,61^ In fact, this gut-lung axis has inspired large randomized controlled trials of gut microbiome modification with the goal of reducing the risk of lung infections (VAP, discussed further below).^62^ Of note, there is also a substantial body of evidence linking airway dysbiosis in ventilated patients with micro- (or macro-) aspiration of oropharyngeal organisms, rather than microbes of the lower gastrointestinal tract.^63–65^ A recent patient-level meta-analysis of microbiome sequencing data from airway secretions of mechanically ventilated patients revealed a large number of differentially abundant taxa in the airway microbiome of patients who developed nosocomial pneumonia versus those who did not, including a number of gut-associated genera including *Enterococcus*, *Bacteroides*, *Faecalibacterium*.^58^ However, machine-learning based selection of factors that predict pneumonia identified reductions in core members of the respiratory microbiome (*Streptococcus*, *Veillonella*) as predictors of pneumonia, rather than enrichment of specific pathobionts.^58^ Overall, further work on the complex microbiology of the lungs is needed to clarify the mechanisms underlying the gut–lung relationship in nosocomial pneumonia.

In addition to taxonomic shifts that transform the gut into a reservoir of potential pathogens, microbiota dysbiosis in critical illness may also lead to an accumulation of antibiotic-resistant organisms, and subsequent risk of infection by antimicrobial-resistant (AMR) pathobionts.^66,67^ A number of studies have found that infections with highly drug-resistant bacteria are associated with increased ICU mortality compared to infections by antibiotic-susceptible organisms.^68–70^ In particular, infections by a common set of highly virulent and antibiotic-resistant pathogens, colloquially called ESKAPE (*Enterococcus spp., S. aureus, K. pneumoniae, A. baumannii, P. aeruginosa*, and *E. coli*), are a leading causes of nosocomial infections worldwide, in addition to being associated with higher economic burden, mortality, and morbidity.^71^ Exposure to antimicrobials, especially those that are active against anaerobes, has been shown to facilitate expansion of antibiotic-resistant pathogens.^72,73^ Suppression of commensal anaerobes by antibiotics results in the loss of both microbe–microbe colonization resistance mechanisms,^74^ as well as host–microbe interactions such as the down-regulation of intestinal C-type lectin RegIIIɣ that keep Gram-positive pathobionts like *Enterococcus* at bay.^75^ However, the contribution of antimicrobial treatments toward AMR in critical illness may depend on the dose, route, and timing of broad-spectrum antibiotic use. For example, studies of patients receiving prophylactic enteral antimicrobials (selective digestive decontamination, SDD) found that AMR gene abundance was not different from patients who did not receiving SDD.^76^ Furthermore, there may be factors beyond antibiotic selective pressure that contribute to the enrichment of AMR pathobionts in the ICU microbiome. For example, recent studies in germ-free and gnotobiotic mice have revealed that AMR bacteria may have a colonization advantage within the gut that is independent of antibiotic pressure. For example, colonization of germ-free mice with 2 strains of *E. coli*, one extensively drug resistant and another that is not, founds that AMR-*E. coli* out-competed and displaced non-resistant *E. coli* in the absence of any antibiotic administration.^77^ Pan-genomic analysis of *E. coli* isolates found that that AMR carriage was associated with diversification of carbohydrate metabolism, providing a potential mechanism underlying gut colonization advantage for drug-resistant *E. coli*.^77^ Overall, gut dysbiosis suffered by ICU patients not only creates a reservoir of pathogens, but also the accumulation of virulent and drug-resistant organisms that contribute to the high incidence, morbidity, and mortality of nosocomial infections in the ICU.

*Pathological microbiome-immune interactions and infections in critical illness –* While most infections in the ICU are from prototypically gut-associated microorganisms, there is not always a clear and direct relationship between gut overgrowth and infecting pathogens. Furthermore, ICU patients are susceptible to a variety of pathogens beyond gut pathobionts, and many infecting microbes in ICU patients do not cause invasive disease in healthy people (such as Coagulase-negative *Staphylococcus*, *Enterococcus*, *Candida* spp. and others).^1^ Instead, this profound vulnerability to a variety of microbes is indicative of a state of compromised immune defenses. Indeed, widespread abnormalities of both innate and adaptive immunity have been observed in critically ill patients, and is known to contribute to their susceptibility to hospital acquired infections.^78–80^ Given the important role of the gut microbiota in shaping immune development and function, together with studies showing pathogen-independent associations between dysbiosis and infections, this has led to the hypothesis that ICU dysbiosis may contribute to nosocomial infections in critical illness via its impact on immune defense mechanisms.

Animal models have elegantly demonstrated the importance of gut microbes in shaping immune development and function.^81^ In addition, animal models have established the detrimental impact of gut dysbiosis on anti-pathogen immune defense mechanisms, both locally in the gut and systemically, which is coupled with impaired survival in mouse models of infection and sepsis.^82,83^ In humans, landmark observational studies have also identified important interactions between the gut microbiota and systemic immunity, including evidence that gut microbiota composition modulates cytokine responses by peripheral blood mononuclear cells,^84^ and guides immune reconstitution after HSCT.^85^ In critically ill patients, longitudinal analysis of microbiota-immune interactions identified that gut *Enterobacteriaceae* enrichment was associated with an increased risk of infection by a diversity of pathogens.^9^ Furthermore, single-cell analysis of blood immune profiles in these patients found that *Enterobacteriaceae* enrichment was coupled with dysregulated mechanisms of innate immune defense, including immature hypofunctional neutrophil populations.^9^ Such findings suggest that the microbiota and immune system are functionally coupled as a “metasystem”, and that dysbiosis of this inter-related metasystem in critical illness renders patients susceptible to diverse pathogens in multiple body compartments.^9^

### 3.2. Dysbiosis and end-organ dysfunction in critical illness

In addition to infections, intestinal dysbiosis has been linked to adverse outcomes in the ICU through its putative contribution to intestinal and extra-intestinal organ dysfunction. Multi-organ dysfunction syndrome (MODS), defined as the failure in two or more organ systems requiring clinical intervention (i.e. mechanical ventilation for respiratory failure, circulatory support for cardiovascular failure, hemodialysis for renal failure, parenteral nutrition for gastrointestinal failure, etc.), is a common and deadly consequence of critical illness.^86^ For many years, the gut has been hypothesized as a “motor” of multi-organ dysfunction. Initially, this hypothesis was based on evidence showing that increased intestinal permeability in ICU patients was associated with, and in fact preceded, multiple organ dysfunction syndrome (MODS).^87^ Additional preclinical data has revealed that loss of gut epithelial barrier integrity led to endotoxin translocation, increased systemic inflammation, culminating in end-organ dysfunction.^83^ In the contemporary era of microbiome science, it is now appreciated that there are multiple cellular and molecular axes of communication between microbes in the gut and extra-intestinal organs.^88^ As such, our understanding of how gut microbes contribute to organ dysfunction in critical illness is becoming more refined. Below we will highlight recent advances in the role of gut dysbiosis in key organ dysfunction syndromes in critical illness, highlighting respiratory failure (acute respiratory distress syndrome, ARDS), and acute neurological dysfunction (delirium/encephalopathy) as some of the best studied organ systems.

*Respiratory failure and ARDS* – A substantial body of research links the gut microbiota to both acute and chronic lung diseases, described as a gut-lung axis.^89^ In critical illness, much of the work on gut-lung axis has focused on the connection between intestinal microbes and acute respiratory distress syndrome (ARDS, a syndrome of acute lung injury and gas-exchange failure that can be precipitated by many common diseases in the ICU including sepsis, trauma, and others) as well as ventilator-associated pneumonia (VAP). The gut-lung axis has been recently reviewed in detail by others,^90^ and therefore will only be briefly summarize here to highlight the role of the gut microbiota in lung dysfunction during critical illness. Seminal work from Dickson and colleagues using both mouse models and ICU patient samples demonstrated that the lung microbiota in ARDS becomes enriched with intestinal microbes (including pathobionts), and this contributes to the propagation of lung inflammation and damage.^61^ Indeed, clinical microbiology of ventilator-associated pneumonia (VAP) in the ICU also reveals a high prevalence of gut-derived pathogens (such as *E. coli*, *Klebsiella*, *Enterobacter*, and others).^1^ The exact route and mechanisms that allow gut organisms to become enriched in the lungs in critical illness are incompletely understood, but may include aspiration of upper GI contents, hematogenous seeding, and changes to the airway microenvironment (eg. inflammation, altered mucous) that favor colonization by pathobionts.^90^ Importantly, the airways contain a homeostatic microbiota that is markedly disrupted in patients with ARDS and VAP. Montassier *et al*. recently characterized lung microbiome signatures that are characteristic of patients with ARDS in the ICU and found that the presence of *Staphylococcus, Ralstonia*, and *Enterococcus* were the best predictors of ARDS.^58^ Therefore, the gut-lung axis in ARDS likely involves complex interplay between injury to the local lung microbiota, relocation of intestinal organisms to the airways, as well as a variety of host factors (immune alterations, airway inflammation, mucous changes) that remain to be fully elucidated.

*Delirium* – Delirium is a nonspecific state of acute neurological dysfunction manifesting as fluctuating confusion and impaired cognition. In the ICU, delirium is associated with increased mortality, duration of ventilation and hospitalization, and long-term cognitive impairment in survivors.^91–93^ Although pathogenesis of delirium is poorly understood, recent studies have highlighted a potential role for gut dysbiosis and abnormal gut-brain axis. Significant differences in gut microbiota composition have been observed between older adults with delirium compared to participants who did not have delirium.^94^ Furthermore, *Enterobacteriaceae* was associated with impaired consciousness and delirium severity.^94^ While work looking into the gut-brain axis in critical illness is limited, an interesting clinical example that sheds light on the potential impact of gut dysbiosis and acute neurological dysfunction is hepatic encephalopathy (HE), which is acute delirium associated with decompensated cirrhosis. Dysbiosis characterized by an overabundance of urease-producing organisms in the gut of cirrhotic patients is thought to create excessive production of ammonia, which spills into the bloodstream causing a state of hyperammonemia.^95^ Ammonia (and possibly other gut-microbe-derived primary or secondary metabolites) may cross the blood–brain barrier, resulting in neuron dysfunction and brain edema.^96^ Interestingly, the treatment for HE is directed at the gut microbiota, through administration of the non-absorbable antibiotic rifaximin as well as nonabsorbable disaccharide lactulose. The example of hepatic encephalopathy provides a compelling link between gut dysbiosis and delirium that can be targeted therapeutically, raising the possibility that other forms of delirium in the ICU may be rooted in pathological gut-brain communication that may be amenable to microbiota-modulating therapies.

In addition to the organ dysfunction in the lung and the brain, gut dysbiosis has been connected with the severity of acute kidney injury,^97,98^ acute and chronic liver injury,^99,100^ coagulopathy (through impacts of dysbiosis on bacterial vitamin K production),^101^ and hematologic abnormalities. However, further research is needed to define the mechanisms underlying the contribution of the microbiota to multiple-organ dysfunction, and guide rationalize therapeutic interventions.

## 4. Microbiome-targeted interventions in critical illness

Evidence linking ICU dysbiosis with adverse outcomes including nosocomial infections, together with the therapeutic malleability of the gut microbiota, has inspired great interest in microbiota modification as a therapeutic adjunct in critical illness. Multiple strategies have been studied in clinical trials, ranging from treatments aimed at depleting the pathogen reservoir with antimicrobials, to treatments aimed at reconstituting “normal” commensals with probiotics or fecal transplantation. Notably, as most evidence positions the microbiota as a proximal driver (or source) of adverse outcomes including nosocomial infections, microbiota interventions in the ICU to date have largely been prophylactic (that is, administered to patients early as prevention against infections or other adverse outcomes). Below, we provide a summary of evidence for microbiome-targeting therapies that have been studied in critically ill humans (an in-depth systematic review of the totality of clinical trial data is beyond the scope of this review), followed by a discussion of current research gaps and strategies to advance the translation of microbiome science in the ICU toward precision microbial therapeutics.

### 4.1. Targeting the intestinal pathogen reservoir with anti-microbials

Evidence implicating altered gut microbiota as a pathogen reservoir in critical illness has led to the hypothesis that “decontaminating” the gut with antimicrobials may reduce infections and mortality in the ICU. To test this hypothesis, studies were conducted testing cocktails of enterally administered, minimally absorbed (low bioavailability) antibacterial agents including polymyxin B, colistin, tobramycin, and/or neomycin, an antifungal agent (amphotericin B), along with a short course of IV cefotaxime. Due to minimal systemic absorption of the enteral agents, this approach was termed “selective digestive decontamination” (SDD), however there are likely effects beyond the GI tract given that systemic antibacterial drugs are also typically used. Microbiota analysis of ICU patients receiving SDD has shown that this treatment decreased pathobionts like *E. coli* and *Candida* burden in the gut, but simultaneously contributes to the loss of commensals including butyrate producers of *Clostridium* clusters IV, XIVa and *F. prausnitzii* .^102,103^ The clinical impact of SDD microbiota modulation has been assessed in multiple large randomized controlled trials, and recent systematic reviews and meta-analyses have concluded that SDD has a favorable impact on nosocomial infections and survival.^104,105^ A more recent, large, randomized controlled trial of SDD in Australian ICUs reported no difference the primary outcome of 90-day mortality between those receiving SDD compared to standard of care, however, there was a reduction in bacteremia as well as a reduction in culturable drug-resistant organisms.^106^ However, concerns over the inconsistent outcomes of SDD trials as well as concerns over its possible contribution to rising rates of drug-resistant microbes has resulted in limited adoption of this practice outside of the Netherlands. Interestingly, despite theoretical concerns about SDD and the risk of AMR, trial data have found that SDD does not appear to increase rates of colonization or infection with AMR pathogens, and on the contrary may be associated with fewer AMR infections,^106–108^ In addition to the gut, antimicrobial-mediated modulation of microbial colonizers in the oropharyngeal tract (selective oropharyngeal decontamination, SOD) has also been evaluated, and is reviewed elsewhere.^105^ Head-to-head comparisons of SDD and SOD have indicated superiority of digestive tract decontamination with respect to hospital and ICU survival.^105^ Nevertheless, the totality of evidence has not led to widespread adoption of SDD in most of the world.

The evidence indicating a reduction of nosocomial infections with SDD underscores the role of the gut microbiota in driving systemic infections in ICU patients. Interestingly, prophylactic antimicrobial therapy outside the gut has also been shown to reduce ICU-acquired infections.^109,110^ Most recently, Ehrmann *et al*. conducted a RCT of inhaled amikacin prophylaxis in mechanically ventilated ICU patients and found that compared to placebo, 3-days of inhaled amikacin reduced the incidence of nosocomial pneumonia.^110^ The underlying mechanism by which inhaled amikacin reduced pneumonia (for example, suppression of specific pathobionts in the lung) and its influence on the lung microbiota were not assessed and raises interesting questions for future research. Overall, depletion of pathogen reservoirs in the microbiota through antibiotic treatments may improve clinical outcomes in the ICU, and the challenge for future research will be to refine the current relatively-broad-spectrum antimicrobial approach toward precision strategies that target pathobionts without eliminating putatively beneficial commensals or driving drug resistance.

### 4.2. Repopulating intestinal commensals with probiotics, prebiotics, and synbiotics

The consistent finding of reduced core microbiome commensals, in particular anaerobic fermenters, in the gut of ICU patients has inspired clinical interventions aiming to repopulate selected commensals. Mechanistically, restoration of core commensals could target both the gut pathogen reservoir by reestablishing colonization resistance against pathobionts, as well as potentially restoring homeostatic microbiota-immune interactions that aid in host defense against infections. Unfortunately, clinical application of probiotic, prebiotic, and synbiotics within randomized controlled trials has been markedly heterogeneous (diverse probiotic strains and prebiotic fibers of varying formulations, doses, and schedules), resulting in a lack of clarity around efficacy that limits their translation into routine clinical practice in the ICU.^111–114^ Furthermore, the choice of probiotic organisms and prebiotic fibers used in this patient population has not been clearly aligned with biological mechanisms. Thus, not surprisingly, most of the largest trials have found these interventions do not improve clinical outcomes in adult patients. For example, the largest randomized controlled trial to date of probiotic therapy in critical illness investigated daily administration of enteral *Lactobacillus rhamnosus GG* versus placebo, and found no difference in rates of nosocomial pneumonia, other infections, nor survival.^62^ The mechanistic rationale for *Lactobacillus rhamnosus GG* as the intervention of choice in this trial is unclear, and unfortunately its impact on the gut microbiota was not studied in trial participants. More detailed understanding of the molecular mechanisms mediating microbiota-immune communication, as well as mechanisms of impaired colonization resistance in the gut during critical illness may highlight particular species (or rationally designed consortia) that are more likely to yield clinical benefit due to keystone roles in these patients. Furthermore, there are unresolved safety concerns with administering live microbes to vulnerable, immunocompromised ICU patients, highlighted by recent reports of *Lactobacillus* bacteremia in 6 of 522 participants treated with *Lactobacillus* probiotics.^115^

In addition to re-populating the gut with live probiotic bacteria, investigators have also begun to evaluate the impact of prebiotics to stimulate the growth and functions of putatively beneficial organisms. Observational data from a prospective cohort of ICU patients found that individuals who received soluble fiber were less acutely ill and less likely to receive broad-spectrum antibiotics, which was linked with higher numbers of SCFA producers including *Blautia* and *F. prausnitzii*.^116^ A synbiotic therapy consisting of a consortia of 7 microbes from the *Streptococcus*, *Lactobacillus*, and *Bifidobacterium* genera together with fructooligosaccharide resulted in a significant reduction in serum endotoxins levels and neutrophil:lymphocyte ratio compared to placebo.^117^ The impact of prebiotic and synbiotic therapies on clinical outcomes have been assessed in multiple, relatively small randomized controlled trials, and a systematic review and meta-analysis of 55 RCTs found that synbiotic therapy was likely superior to prebiotics alone with respect to the outcome of nosocomial infections.^112^ However, there was no evidence indicating that they reduce hospital and ICU mortality.^112^ While investigations have indicated that prebiotic and synbiotic treatments yield varying effects on outcomes, it is important to note the significant methodological variations across studies likely contributes to the lack of uniformity in results.

### 4.3. Fecal microbiota transplant

Studies using FMT in critically ill patients have been limited to case reports/series, and thus are heavily susceptible to bias. A limited number of cases of FMT in critically ill patients with multi-system disease have reported dramatic clinical improvements in recipients including reduction in fever and systemic inflammatory response within days, and an immediate improvement in diarrhea.^118–121^ A case series of 18 ICU patients who underwent rescue FMT for intractable antibiotic-associated diarrhea reported that 13 out of 15 patients achieved improvement in diarrhea.^122^ The biological impact of FMT on microbial communities of the gut (and other organs) in ICU patients has not been explored. It will also be important for future studies to address concerns around the safety of FMT in severely ill and immunocompromised patients, underscored by recent high-profile reports of infection by drug-resistant pathogens associated with FMT.^123^ DeFilipp and colleagues identified 2 immunocompromised patients enrolled in FMT trials who developed bacteremia (one fatal) caused by extended-spectrum beta-lactamase producing *E. coli*, which was traced back to the same donor stool as the likely source.^123^ Despite these reports, it is likely that the risk of donor-stool-derived infections in FMT are low, even in immunocompromised patients. For example, in a cohort of 22 HSCT recipients with acute graft-versus-host disease and high rates of infections, metagenomic analysis of host and donor microbiota as well as infecting pathogen did not find any evidence that infections originated from donor stool.^124^ However, the impact of FMT on gut permeability and translocation of endogenous host-derived pathobionts in acute and critically ill patients remains to be defined. Collectively, FMT remains very uncommon in the ICU, and its potential role in this patient population is uncertain.

## 5. Key areas for future research

Despite the exciting and impactful research that has emerged in recent years, much remains to be learned about the ICU microbiome. Nearly all ICU microbiome studies to date have focused on bacterial communities, yet it is increasingly apparent that other kingdoms of microorganisms such as fungi and viruses play important roles in the ecology of the gut, as well as underappreciated contributions to microbiome–host interactions. Haak and colleagues used a combination of marker gene sequencing for bacteria and fungi (16S and ITS1 genes, respectively) as well as virus discovery cDNA-amplified fragment length polymorphism next-generation sequencing (VIDISCA-NGS) to describe the multi-kingdom composition of 33 critically ill patients.^22^ They found that pathobiont bacteria including *Staphylococcus, Enterococcus, Klebsiella, Escherichia* and *Enterobacter* tend to co-occur with fungal members like *Candida, Aspergillus* and *Debaryomyces*, as well as bacteriophages that target these bacteria.^22^ Abbas *et al*. discovered a new family of circular DNA viruses, *Redondoviridae*, that maybe enriched in the respiratory tract in ICU patients compared to healthy controls.^125^ A recent study of patients with severe COVID-19 identified fungal dysbiosis in the GI tract characterized by *Candida* overgrowth, which was coupled to adverse reprogramming of myeloid progenitor cells and altered neutrophil responses that worsened lung inflammation.^126^ Clearly, ICU dysbiosis extends beyond the bacterial microbiome, but much remains to be discovered about the functional contributions of non-bacterial microbes toward the pathogenesis of critical illness. A key challenge for future research will be to develop a deeper and more comprehensive understanding of the multi-kingdom ecology of ICU dysbiosis, the inter-kingdom dynamics, and their role in immune dysregulation, infections, and other outcomes in critical illness.

An additional challenge for the ICU microbiome research community will be to translate observations linking dysbiosis and adverse outcomes into rationalized and targeted therapeutic interventions. We propose that a crucial ingredient in the success of microbiota-targeted interventions in the ICU involves moving away from empirical and untargeted interventions (generic probiotics, broad-spectrum antibiotics, FMT) toward mechanism-guided approaches that target specific mediators of microbiota–host interactions. For this, further emphasis is needed on defining causal mechanisms mediating the relationships between dysbiosis and outcomes, as well as understanding the mechanistic impacts of microbiome-targeting interventions in the setting of clinical trials. This will likely require an integrative multi-omics approach when studying patient samples, as has been used recently to identify potentially important microbiota-immune dysregulation in nosocomial infections.^9^ Furthermore, complementing human studies with reverse-translational models such as humanized gnotobiotic mice, wherein human microbiomes are engrafted in germ-free mice, has proven to be a powerful strategy to dissect cellular and molecular mechanisms of microbiome–host interactions in other diseases,^127–129^ and can be very useful for pre-clinical testing of targeted microbial therapeutics prior to human clinical trials.^128^ However, it is important to acknowledge that germ-free mice possess a markedly altered immune system compared to conventionally raised mice, which must be considered in the development of therapeutics targeting human diseases that have a large immune component, such as sepsis.

Lastly, the translation of microbiome science into effective targeted therapies in the ICU will require that we address a complex issue in personalized microbiome medicine – heterogeneity – of both microbiota as well as host factors. While population/cohort-level signatures of ICU dysbiosis have emerged, the quest for personalized microbiome therapies in the ICU will require applying our learnings to individual patients. Therefore, further scrutiny and validation of signatures of dysbiosis at the level of individual patients is essential, and will require improved microbiome-related diagnostics. Common sequencing methods such as amplicon-based or shotgun sequencing are unsuitable for rapid point-of-care diagnostics due to their time-consuming workflow, multiplexed design, and cost. Culture-based techniques may be employed for detecting microorganisms of interest, but are also time-consuming and only identify a limited number of microorganisms. Alternatively, next-generation long read sequencing methods are relatively cost-effective and may be a viable method for rapid microbiome diagnostics.^130^

Beyond the microbiome, there is also substantial heterogeneity of clinical variables between individual patients that may impact a patient’s response to microbial therapeutics in the ICU.^16^ Factors such as age, sex, comorbidities, and underlying diagnosis causing critical illness will need to be considered together with microbiota phenotype. For example, basic patient factors such as biological sex are known to interact with the microbiome in a manner that may influence the response to microbial therapeutics. Microbial communities in the gut have been found to differ between males and females, and these sex-based microbiome differences can influence sexual dimorphism of immune responses in disease.^131^ Preclinical studies using mouse models of autoimmune diabetes have found that the incidence and early-onset of diabetes is greater in females compared to males, but this difference was abolished in GF mice.^132^ Furthermore, male-to-female microbiota transfer protected females from early-onset diabetes, suggesting that the combination of biological sex and microbiome may have an important influence on disease pathogenesis as well as response to therapy.^132,133^ In critical illness, sex-biased clinical outcomes are common,^134^ and gut microbiota differences have been demonstrated in ICU patients. Therefore, further work is needed to disentangle the relationships between important patient factors such as sex, microbiome dysbiosis, and mechanisms of disease pathogenesis in order to embrace the need for a personalized approach to precision microbial therapies in the heterogeneous realm of critical care.

## Data Availability

This review article does not present any new data.

## List of abbreviations

ICU: intensive care unit
AMR: antimicrobial resistance
CR: colonization resistance
SDD: selective digestive decontamination
SDD: selective oropharyngeal decontamination
SCFA: short chain fatty acids
VAP: ventilator-associated pneumonia
ARDS: adult respiratory distress syndrome
BSI: bloodstream infection
HSCT: hematopoietic stem cell transplant
